# Combined Transcriptome and Metabolome Profiling Provide Insights into Cold Responses in Rapeseed (*Brassica napus* L.) Genotypes with Contrasting Cold-Stress Sensitivity

**DOI:** 10.3390/ijms232113546

**Published:** 2022-11-04

**Authors:** Xinhong Liu, Ran Wei, Minyu Tian, Jinchu Liu, Ying Ruan, Chuanxin Sun, Chunlin Liu

**Affiliations:** 1 Key Laboratory of Hunan Provincial on Crop Epigenetic Regulation and Development, Hunan Agricultural University, Changsha 410128, China; 2 Crop Research Institute, Hunan Academy of Agricultural Sciences, Changsha 410125, China; 3 Key Laboratory of Crop Physiology and Molecular Biology of Ministry of Education, Hunan Agricultural University, Changsha 410128, China

**Keywords:** cold-stress tolerance, ROS scavenging, silique length, sugar metabolism, ABA signaling, JA signaling, MAPK signaling, photosystems

## Abstract

Low temperature is a major environmental factor, which limits rapeseed (*Brassica napus* L.) growth, development, and productivity. So far, the physiological and molecular mechanisms of rapeseed responses to cold stress are not fully understood. Here, we explored the transcriptome and metabolome profiles of two rapeseed genotypes with contrasting cold responses, i.e., XY15 (cold-sensitive) and GX74 (cold-tolerant). The global metabolome profiling detected 545 metabolites in siliques of both genotypes before (CK) and after cold-stress treatment (LW). The contents of several sugar metabolites were affected by cold stress with the most accumulated saccharides being 3-dehydro-L-threonic acid, D-xylonic acid, inositol, D-mannose, D-fructose, D-glucose, and L-glucose. A total of 1943 and 5239 differentially expressed genes were identified from the transcriptome sequencing in XY15CK_vs_XY15LW and GX74CK_vs_GX74LW, respectively. We observed that genes enriched in sugar metabolism and biosynthesis-related pathways, photosynthesis, reactive oxygen species scavenging, phytohormone, and MAPK signaling were highly expressed in GX74LW. In addition, several genes associated with cold-tolerance-related pathways, e.g., the CBF-COR pathway and MAPK signaling, were specifically expressed in GX74LW. Contrarily, genes in the above-mentioned pathways were mostly downregulated in XY15LW. Thus, our results indicate the involvement of these pathways in the differential cold-stress responses in XY15 and GX74.

## 1. Introduction

The rapeseed (*Brassica napus* L.) (AACC, 2n = 38) is a multipurpose crop which originated from natural hybridization between *B. campestris* (AA, 2n = 20) and *B. oleracea* (CC, 2n = 18) [1,2]. It is a major oilseed crop that accounted for 14% of the 205 million metric tonnes of world vegetable oil consumed in the 2020/2021 marketing year (http://www.soystats; accessed on 21 September 2022). It is highly valued by consumers due to its high unsaturated fatty acid and protein contents, which is why it is used as a vegetable for humans and fodder for animals. Its use in biofuel production further increases its economic importance [3]. It is ranked second to the soybean in terms of world protein meal consumed in the 2020/2021 marketing year (http://www.soystats; accessed on 21 September 2022).

Due to its economic importance, intensive breeding efforts have been put in place to increase rapeseed yield and quality [2,4,5,6]. This crop is mostly cultivated as a winter or semi-winter form in Europe and Asia, whereas spring-sown rape types are more suitable to the climatic conditions in Canada, northern Europe, and Australia [5]. One of the features of semi-winter in China is the unpredictable temperature, which can be within the range of −38 to −4 °C [7], which proves to be a threat to rapeseed production [8]. Cold stress increases branching and prolongs the flowering period in rapeseed [9]. More pods are produced under cold stress but most are poorly filled, leading to a 10.90% yield reduction [9,10,11]. In southern China, rice–oilseed and rice–rice–oilseed crop rotation productions require the planting of early-maturing and extra-early-maturing rapeseed varieties. The growth process of early-maturing rapeseed cultivars is fast, and the flowering period is early. During these periods, once exposed to freezing or continuous low temperature and rainy weather, the young silique easily freezes and even completely dies, leading to yield reduction and poor seed quality [10,12,13]. Thus, it is important to understand the cold-induced molecular responses in rapeseed.

Among the major genes related to cold-stress tolerance, Lee et al. [14] discovered the inducer of the *CBF* expression 1 (*ice1*) gene in Arabidopsis. They found that a mutant defective in an upstream transcription factor is required for chilling and freezing tolerance largely under plant hormonal regulation. This gene is a part of the CBF-COR pathway, where CBF (C-repeat-binding factor) regulates the expressions of many cold-responsive (COR) genes [15]. In addition to this pathway, a number of carbohydrates and polyamines were identified as cold-responsive metabolites in peanuts, and several key genes involved in soluble sugar, polyamine, and G-lignin biosynthetic pathways were substantially higher and/or responded more quickly in cold-tolerant peanuts as compared to cold-sensitive peanuts under low temperature [16]. So far, in *Brassicas*, Mehmood et al. [17] identified 138 and 55 differentially expressed genes (DEGs) and proteins, respectively, in two contrasting rapeseed lines. The detected DEGs were largely involved in photosynthesis, antioxidant enzymes, and energy metabolism; from this, three homolog genes (*BnCML, BnCAT,* and *BnNIR*) were verified in the freezing response [17]. Moreover, Sinha et al. [18] applied de novo transcriptome profiling of cold-stressed siliques during pod-filling stages in Indian mustard (*B. juncea* L.) and found that transcription regulators and protein kinases were induced only during early silique development. Raza et al. [19] have profiled cold stress-responsive genes, metabolites, and metabolic pathways to understand cold-stress tolerance and susceptibility in two contrasting genotypes of rapeseed. Their study revealed that most of the DEGs and differentially accumulated metabolites were involved in starch and sucrose metabolism as well as phenylalanine metabolism. Nevertheless, the studies by Raza, Su, Hussain, Mehmood, Zhang, Cheng, Zou, and Lv [19] and Jian et al. [20] used leaf samples from 28-week old seedlings and reported several cold-responsive genes/pathways. However, a leaf-based analysis may not be a true reflection of transcriptional and metabolite changes in a maturing rapeseed at podding stage. In recent years, multi-omics approaches have emerged as successful technologies for plant systems biology and have enhanced our understanding of molecular regulatory networks controlling biotic and abiotic stress responses [21,22].

Considering the limited availability of the knowledge about cold-responsive metabolites, genes, and pathways in rapeseed siliques, the present study explored the cold-stress responses in siliques of two rapeseed genotypes with contrasting cold responses, i.e., XY15 (cold-sensitive) and GX74 (cold-tolerant). We used the combined approach of physiological performance, transcriptome sequencing, metabolome profiling, and quantitative real-time polymerase chain reaction (qRT-PCR) analyses to explore the key genes and pathways that are activated in response to cold stress. Through our comparative analyses, we discussed the role of the CBF-COR pathway, soluble sugars/saccharides, photosynthesis, antenna proteins, chlorophyll biosynthesis, reactive oxygen species (ROS) scavenging, ABA, JA, and MAPK signaling, and transcription factors in cold-stress tolerance in rapeseed siliques. The findings from this study would be useful for breeding cold-tolerant rapeseed, while optimizing the pod/seed yield to meet the nutritional and industrial needs.

## 2. Results

### 2.1. Physiological Performance of Two Contrasting Rapeseed Genotypes to Progressive Cold Stress

**Figure 1:**
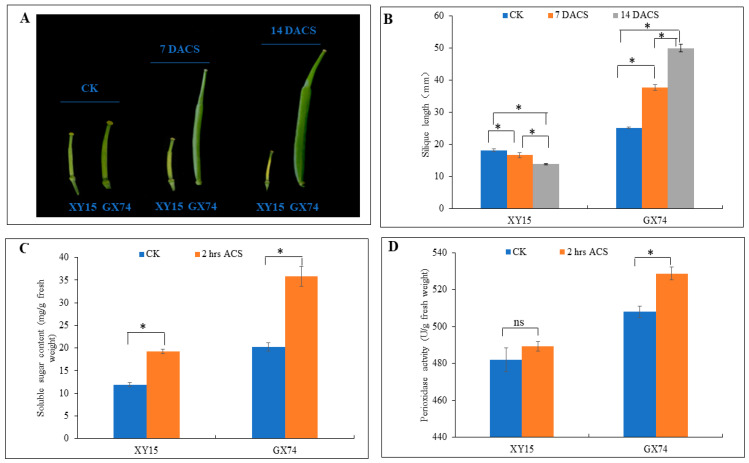
Physiological performance of XY15 (cold-sensitive) and GX74 (cold-tolerant) rapeseed genotypes. (**A**) Silique before cold stress (CK), and 7 and 14 days after cold stress (DACS), respectively. (**B**) silique length. (**C**) Soluble sugar content. (**D**) Peroxidase activity between CK and 2 h after cold stress (ACS). Error bars represent standard error of means. Bars with * indicate significant differences (by two-tailed *t*-test at *p* ≤ 0.05), while ns indicates no significant difference between the sampling periods.

Two rapeseed genotypes, i.e., XY15 (sensitive) and GX74 (tolerant), with contrasting cold-stress tolerance were assessed for their physiological traits before and after cold-stress treatment (−2 °C). The XY15 genotype had yellow and wilted silique at 7 and 14 days after cold stress (DACS) (Figure 1A). The silique length of the XY15 genotype before cold stress (CK) decreased steadily and significantly (*p* < 0.05) at 7 and 14 DACS. In contrast, cold stress did not affect the silique growth in the GX74 genotype as it increased significantly (*p* < 0.05) after cold stress (Figure 1B). These observations indicate that silique growth is significantly impaired in XY15 as compared to GX74 under cold stress. Cold stress provoked an increase in total sugar in the two genotypes, but we observed a stronger sugar accumulation upon cold stress in the tolerant genotype (Figure 1C), suggesting that sugar biosynthesis related pathways might be affected in XY15. Although the peroxidase (POD) activity increased slightly after cold stress application in XY15, it did not differ statistically (*p* > 0.05) (Figure 1D). Contrastingly, we observed a significantly higher induction of peroxidase activity in GX74 when treated with cold stress. We deduce that the ROS scavenging ability of the XY15 genotype is lower as compared to GX74. Thus, based on these observations, we can hypothesize that cold sensitivity in XY15 is possibly related to changes in growth- and development-related, sugar biosynthesis, and ROS-scavenging-related genes/pathways.

### 2.2. Metabolome Profiles of XY15 and GX74 Siliques in Response to Cold-Stress Tolerance

**Figure 2:**
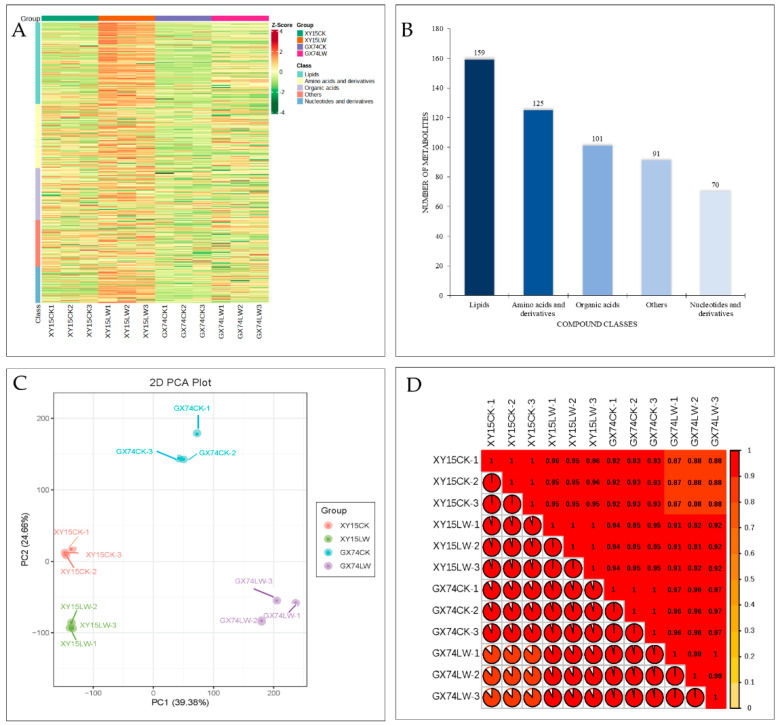
Metabolome analysis of rapeseed siliques treated with cold stress. (**A**) Heatmap of the relative intensities of the accumulated compounds. (**B**) Compound classification. (**C**) Principal component analysis. (**D**) Pearson’s correlation coefficient of the relative intensities of the compounds accumulated in rapeseed siliques in response to cold-stress treatment. Where XY15 and GX74 are cold-sensitive and -tolerant genotypes, respectively, and CK and LW are control and cold treatment, respectively. The numbers with the treatments represent the replicates.

In order to understand the changes induced by cold stress in the silique samples of the two genotypes, we performed widely targeted metabolome profiling under cold stress and CK. In total, we detected 545 metabolites (Figure 2A). These metabolites could be classified as lipids (29%), amino acids and derivatives (23%), organic acids (18%), others (17%), and nucleotide and derivatives (13%) (Figure 2B). The large proportion of lipid compounds highlights their relevance in regulating rapeseed’s response to cold stress. The PCA showed the grouping of replicates of the same variety and treatment (Figure 2C), indicating that the sampling was reliable. This was further confirmed by the observation that PCC was >0.84 (Figure 2D). Of the 545 metabolites, 156 were differentially accumulated in the two genotypes. These differentially accumulated metabolites (DAMs) were screened based on the partial least squares-discriminant analysis (PLSDA) with a threshold of log2FC ≥ 1 and variable importance of the projection (VIP) ≥ 1. Compared to the respective controls, 96.95 and 86.41% of the compounds showed increased accumulations after cold-stress treatment in XY15 and GX74, respectively (Table S2).

#### 2.2.1. Metabolome Analysis Indicates Increased Accumulation of Saccharides in Rapeseed Siliques in Response to Cold Stress

Sixty-eight detected metabolites classified as saccharides enriched in 32 different KEGG pathways were variedly accumulated in XY15 and GX74 in studied cold conditions (Table S2). The sum of all the saccharides decreased in XY15LW as compared to XY15CK, whereas it increased in GX74LW as compared to GX74CK, thus confirming the observed changes in total soluble sugar (TSS) in response to cold stress. The most accumulated saccharides in rapeseed siliques in response to cold stress were 3-dehydro-L-threonic Acid, D-xylonic acid, inositol, D-mannose, D-fructose, D-glucose, and L-glucose. However, their relative contents were higher in GX74LW as compared to XY15. Overall, 31 saccharides showed reduced accumulation in XY15LW as compared to XY15CK, whereas 47 saccharides were increasingly accumulated in GX74LW as compared to GX74CK (Table S3). These observations clearly indicate that sugar biosynthesis is increased in rapeseed siliques in response to cold stress in both genotypes, whereas the tolerance in GX74 can be associated with relatively higher saccharides content.

#### 2.2.2. Lipid Biosynthesis (Free Fatty Acids and Glycerol) and Amino Acids and Derivatives Are Increased in Rapeseed Siliques in Response to Cold Stress

Eighty-seven metabolites classified as lipids (free fatty acids and glycerol esters) were differentially accumulated in the studied silique samples of the two varieties. Overall, 84 and 80 lipids were increasingly accumulated in XY15 and GX74, respectively, as compared to their respective controls. Interestingly, 10-hydroxydecanoic acid was not accumulated in the control of either variety and only accumulated after cold-stress treatment. Similarly, myristoleic acid accumulated only in GX74LW. Overall, we observed that lipids accumulated in higher quantities in XY15 as compared to GX74 except 10-hydroxydecanoic acid, docosanoic acid (behenic acid), and arachidic acid (Table S2). These observations generally support earlier reports that increasing the contents of lipids/fatty acids in the membrane lipids can enhance cold resistance in low-temperature environments [23,24]. Similar to the lipids, the amino acids and their derivatives’ contents increased in rapeseed siliques after cold-stress treatment, indicating that cold induces their biosynthesis. We observed that the relative contents of the amino acids and their derivatives were higher in cold-treated XY15 as compared to GX74. However, interestingly, we found that N-methyl-L-glutamate and phenylacetyl-L-glutamine were accumulated in higher quantities in GX74LW as compared to XY15LW.

#### 2.2.3. Cold Stress Induces Changes in Nucleotides and Derivatives and Organic Acids of Rapeseed Siliques

The comparative metabolome profile showed differential accumulations of 29 metabolites classified as nucleotides and derivatives. Overall, the accumulation of these compounds increased in response to cold-stress treatment in both genotypes; GX74LW had higher contents as compared to XY15LW. The most interesting observation was the accumulation of 8-hydroxyguanosine in response to cold stress in both genotypes, where it was not detected in non-stress siliques. We observed that 9-(arabinosyl)hypoxanthine, inosine, uridine, guanosine 3′,5′-cyclic monophosphate, and β-pseudouridine were the most up-accumulated nucleotides and derivatives in response to cold stress (Table S2). The up-accumulation of 8-hydroxyguanosine, adenine, riboprine, cytidine, and N6-isopentenyladenine in GX74LW as compared to XY15LW, together with overall increased accumulation of nucleotides and derivatives, could be a cold-stress-tolerance response in GX74.

Fifteen compounds classified as organic acids were differentially accumulated in the two varieties in response to cold-stress treatment. The highest log2 foldchange was observed for 4-Acetamidobutyric acid, followed by phenylpyruvic acid, 6-hydroxyhexanoic acid, valeric acid, γ-aminobutyric acid, and oxalic acid in both genotypes. In addition, we noted the increased accumulation of Jasmonic acid in response to cold-stress treatment in both genotypes. α-Ketoglutaric acid was down-accumulated in XY15LW as compared to XY15CK, whereas it was up-accumulated in GX74CK as compared to GX74LW. This compound is involved in ascorbate and aldarate metabolism and could be responsible for relatively higher peroxidase content (Figure 1D). Interestingly, in addition to JA, jasmonoyl-L-isoleucine, N6-isopentenyladenine, and abscisic acid exhibited increased accumulation in GX74LW as compared to GX74CK. Contrastingly, N6-Isopentenyladenine and ABA showed down-accumulation in XY15LW as compared to XY15CK. Overall, these observations present the compounds that could be associated with cold-stress tolerance in GX74 and cold sensitivity in XY15.

Similar to the lipids, the amino acids and their derivatives’ contents increased in rapeseed siliques after cold-stress treatment, indicating that cold induces their biosynthesis. We observed that the relative contents of the amino acids and their derivatives were higher in cold-treated XY15 as compared to GX74. However, interestingly, we found that N-methyl-L-glutamate and phenylacetyl-L-glutamine were accumulated in higher quantities in GX74LW as compared to XY15LW, implying that glutamate is contributing to cold tolerance in the former as compared to the latter.

Overall, these differences in metabolite profiles indicate their involvement in cold susceptibility or tolerance. In addition, these changes suggest the role of the presented pathways in silique growth differences.

### 2.3. Transcriptome Profiles of Cold-Treated Siliques of XY15 and GX74

#### 2.3.1. Overview of Transcriptome Sequencing Data

We further performed a transcriptome analysis to get insight into the gene expression changes induced by cold stress and highlight key pathways as well as differentially expressed genes. The sequencing of 12 libraries (two genotypes * two conditions * three replicates) produced an average of 49,198,739 raw reads (47,559,973 to 51,782,717) (Table S4). More than 90% of the total reads were successfully mapped to the reference genome “Darmor-bzh” [1], of which at least 86.30% were uniquely mapped with an error rate ≤0.03. A total of 87,969 genes comprising 79,613 known and 8356 novel genes were detected from the 12 libraries. The fragments per kilobase of exon per million fragments mapped (FPKM) distribution of the expressed genes showed that the genes had relatively different expression profiles among the samples (Figure S1A). The PCA and PCC (r ≥ 0.87) further confirmed that sampling was reliable (Figure S1B,C). Thus, both the metabolome and transcriptome results correlate in this regard. Of the 87,969 genes detected, 85,465 genes were successfully annotated to nine public databases (Figure S1D). To further confirm RNA-seq data, we selected 20 genes and profiled their expression using the qRT-PCR approach. The relative expression levels of the selected genes in qRT-PCR were consistent (R^2^ = 86.92%) with the RNA-seq (Figures S2 and S3). This gives further credence to the reliability of our RNA-seq results.

#### 2.3.2. Key Differential Expression Changes Related to Cold-Stress Treatment

**Figure 3:**
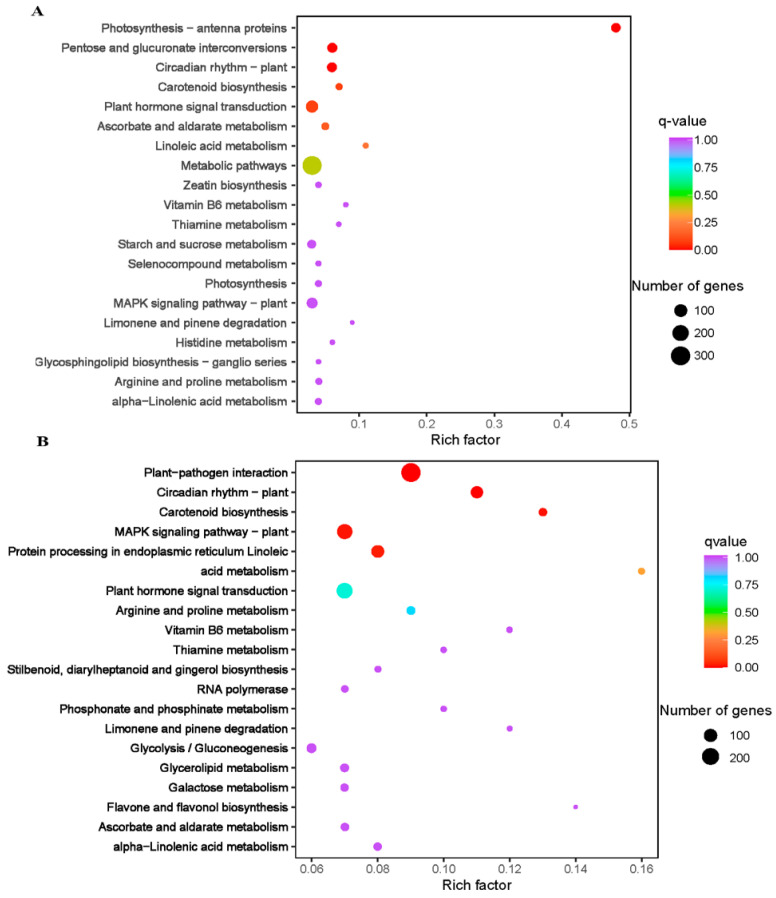
Kyoto encyclopedia of genes and genomes’ pathway enrichment scatter bar plots of differentially expressed genes in the two contrasting genotypes of rapeseed (XY15 (sensitive) and GX74 (tolerant)) under before (CK) and after cold treatment (LW). (**A**). XY15CK vs. XY15LW. (**B**). GX74CK vs. GX74LW.

Based on the criteria of log2 foldchange (log2FC) ≥ 1 and false discovery rate (FDR) with the adjusted *p*-value (padj) < 0.05, a total of 6254 DEGs were screened. Totals of 1943 and 5239 genes were differentially expressed in XY15CK_vs_XY15LW and GX74CK_vs_GX74LW, respectively (Figure S4A). Only 928 out of 6254 DEGs were commonly expressed in the two genotypes (Figure S4B). Most of the DEGs were upregulated in cold-treated siliques, indicating that cold stress induced an increase in their expression in both genotypes. Additionally, we observed that the tolerant genotype (GX74) had a nearly three-times higher number of DEGs than the sensitive genotype (XY15). This difference may account for the difference in response to cold stress. KEGG pathways enrichment showed that DEGs were enriched in different development and signaling pathways, i.e., MAPK signaling, plant-hormone signal transduction, glycolysis/gluconeogenesis, photosynthesis—antenna proteins, pentose, circadian rhythm—plant, and carotenoid biosynthesis (Figure 3C,D).

**Transcriptome analysis confirms physiological and metabolomic results related to sugar biosynthesis**

**Figure 4:**
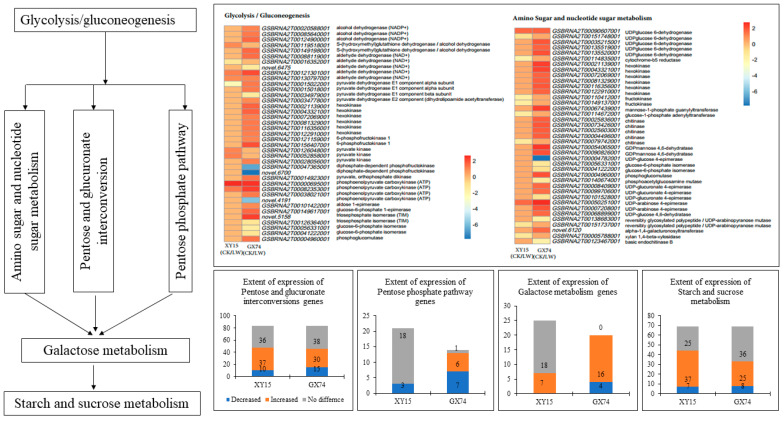
Sugar-related metabolism pathways in XY15 (cold-sensitive) and GX74 (cold-tolerant) rapeseed siliques in response to cold treatment. The heatmaps represent the log2 foldchange values of genes in glycolysis/gluconeogenesis, and amino sugar and nucleotide sugar metabolism. The lower four bar graphs show the other four interconnected pathways and extent of gene expression in the two genotypes before and after cold stress. The expression profiles of the genes are shown in Table S5.

The physiological and metabolome analyses indicated an increase in TSS and saccharides in response to cold-stress treatment in both XY15 and GX74 siliques; therefore, we studied the expression changes in respective genes/pathways. In addition, the KEGG pathway enrichment analysis showed that DEGs in both genotypes were enriched in pathways such as Glycolysis/Gluconeogenesis, amino sugar and nucleotide sugar metabolism, galactose metabolism, pentose and glucuronate interconversions, pentose phosphate pathway, and starch and sucrose metabolism (Figure 4). The amino sugar and nucleotide sugar pathway is present upstream of all of these pathways; therefore, we first explored transcriptomic changes in it. Overall, 41 transcripts annotated as 19 genes were differentially expressed; of these only 11 were expressed in XY15, whereas 34 were GX74-specific (Figure 4; Table S5). The higher accumulations of D-glucose 6-phosphate, D-fructose 6-phosphate, and D-mannose are consistent with the increased expressions of hexokinases (HKs) and phosphoglucomutase (PGM). Similarly, the D-xylose and D-galactose contents are consistent with the expression changes in xylan 1,4-beta-xylosidase (XYL4) and UDP-glucose 4-epimerase (galE). An associated pathway, which is linked with the amino sugar and nucleotide sugar pathway, i.e., pentose and glucuronate interconversions, showed enrichment of 44 and 33 transcripts in XY15CK vs. XY15LW and GX74CK vs. GX74LW, respectively. Most of the pectinesterases (PE), galacturan 1,4-alpha-galacturonidase (GalAG), and pectate lyases showed increased expression after cold-stress treatment in XY15 (Figure 4; Table S5). Along with these DEGs, alcohol dehydrogenase (NADP+), UDPglucose 6-dehydrogenase, and polygalacturonase (PGs) were upregulated in GX74LW as compared to GX74CK as well as XY15LW. The expression of these genes is consistent with the accumulation patterns of D-galacturonic acid, which affects the downstream pathway, i.e., amino sugar and nucleotide sugar metabolism. Totals of 9 and 36 transcripts were enriched in glycolysis/gluconeogenesis in XY15CK vs. XY15LW and GX74CK vs. GX74LW, respectively (Figure 4; Table S5). Of the nine transcripts, S-(hydroxymethyl)glutathione dehydrogenase/alcohol dehydrogenase, pyruvate kinase (PK), and phosphoenolpyruvate carboxykinase (ATP) (pckA) were upregulated in XY15LW as compared to XY15CK. These DEGs are present downstream from the citrate cycle and are involved in pyruvate and acetaldehyde biosynthesis. Contrarily, other than these DEGs, several genes regulating the biosynthesis of α-D-glucose, α-D-glucose 6p, β-D-glucose, β-D-glucose 6p, and β-D-fructose 6p were exclusively expressed in GX74 and their expressions increased after cold-stress treatment. Finally, the upregulation of DEGs enriched in starch and sucrose metabolism, i.e., PEC, GalAG, UDPglucose 6-dehydrogenase, and PL, increase the saccharides content in XY15 after cold treatment (Table S5). Whereas, the exclusive expressions of a larger number of transcripts of the same genes as well as alcohol dehydrogenase (NADP+) and PEs enable GX74 to have higher cold-stress tolerance (Table S5). Thus, these and other exclusively expressed genes in GX74 are responsible for higher saccharides (and TSS) biosynthesis, whereas their non-expression in XY15 renders it cold-sensitive.

Genes related to photosynthesis, antenna proteins, and chlorophyll biosynthesis are downregulated in rapeseed siliques in response to cold stress

**Figure 5:**
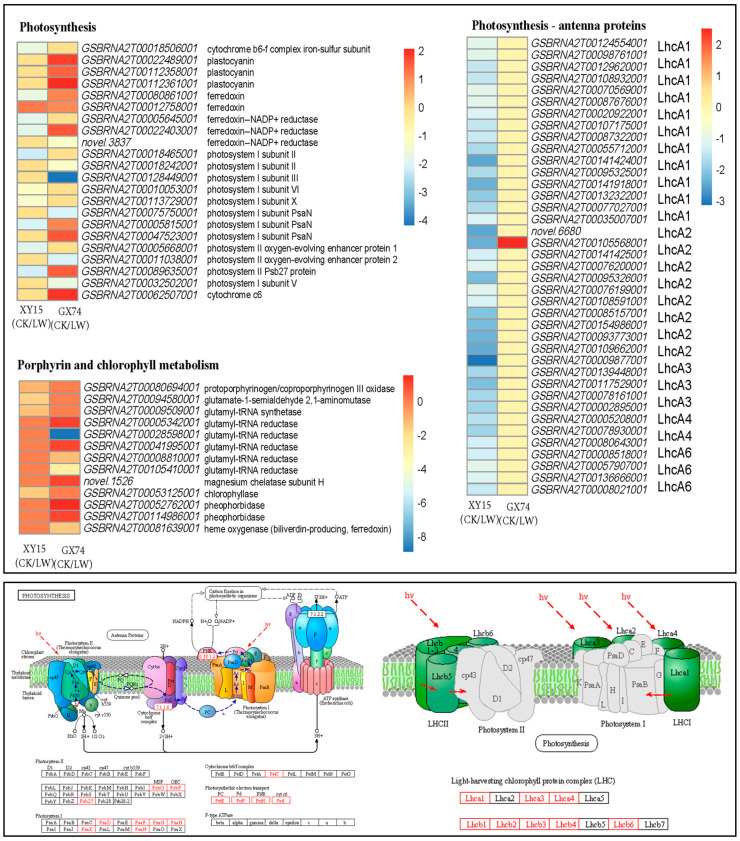
Differential regulation of photosynthesis, photosynthesis—antenna proteins, and chlorophyll-biosynthesis-related genes in XY15 (cold-sensitive) and GX74 (cold-tolerant) rapeseed siliques in response to cold treatment. The heatmaps represent the log2 foldchange values. The lower two panels showing photosynthesis as well as antenna pathways were generated using KEGG pathway database [25]. The differentially expressed genes are highlighted as red text. CK and LW represent control and cold treatments. The gene name abbreviations refer to the full names presented in Table S5.

Interestingly, we observed that a large number of transcripts (39) annotated as eight photosynthesis—antenna proteins were differentially expressed in XY15CK vs. XY15LW. These included three photosystem I (PSI) proteins, i.e., light-harvesting complex I chlorophyll a/b binding proteins (LhcA1, LhcA3, and LhcA4), and five photosystem II (PSII) proteins, i.e., light-harvesting complex II chlorophyll a/b binding proteins (LhcB1, LhcB2, LhcB3, LhcB4, and LhcB6). All these transcripts were downregulated in XY15LW as compared to XY15CK, indicating that the antenna complex of this genotype is highly damaged under cold stress. Contrarily, only LhcB1 was differentially regulated (upregulated) as a result of cold stress in GX74. These observations indicate that cold stress significantly damages PSI and PSII antenna proteins in XY15 but not in GX74, thus enabling the latter to tolerate the cold stress (Figure 5; Table S5).

Similar expression trends, i.e., downregulation after cold treatment in XY15, were observed for the genes enriched in photosynthesis. However, several genes (or some of their transcripts) such as plastocyanin (petE), ferredoxin (petF), ferredoxin--NADP+ reductase (petH), photosystem I subunit PsaN (PsaN), photosystem II Psb27 protein (Psb27), and cytochrome c6 (petJ) were upregulated in GX74LW as compared to GX74CK. These changes indicate that the photosynthesis is also disturbed in XY15, whereas it is not disturbed to the same extent in GX74. These observations are consistent with the observed morphology in both genotypes (Figure 1A). Additionally, we observed a yellowing of the siliques in XY15, whereas those of GX74 remained green; therefore, we checked the expression changes in chlorophyll biosynthesis. All the transcripts in porphyrin and chlorophyll biosynthesis metabolism were downregulated in XY15LW as compared to XY15CK, especially chlorophyllase. Whereas, in the case of GX74, some transcripts of glutamyl-rRNA reductase (gltX), magnesium chelatase subunit H (chlH), and pheophorbidase (PPD) were upregulated in response to cold treatment (Figure 5; Table S5).

**Peroxidase and flavonoid biosynthesis genes are upregulated in XY15 and GX74 in response to cold stress**

**Figure 6:**
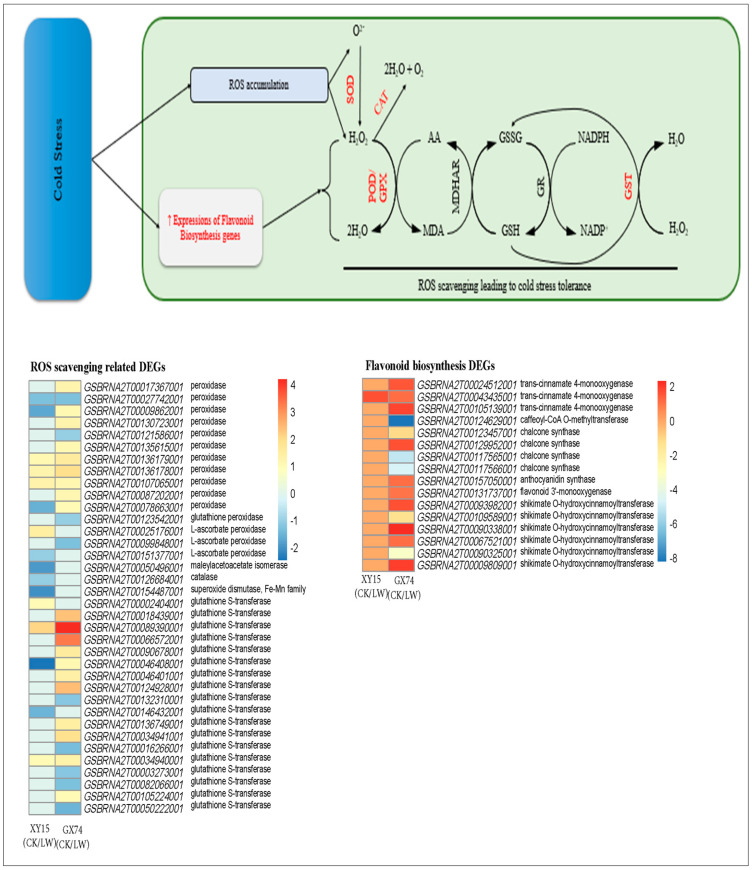
Reactive oxygen species scavenging related gene expression changes in XY15 (cold-sensitive) and GX74 (cold-tolerant) rapeseed siliques after cold-stress treatment. The genes highlighted as red text were differentially expressed. GR: glutathione reductase; MDHAR: monodehydroascorbate reductase; GPX: glutathione peroxidase; SOD: superoxide dismutase; CAT: catalase; GSH: reduced glutathione; GSSG: oxidized glutathione; MDA: monodehydroascorbate; and AA: ascorbate (AA). The heatmap was drawn in Tbtools [27] using the log2 foldchange values of ROS-scavenging- and flavonoid-biosynthesis-related genes. CK and LW represent control and cold treatments. The gene name abbreviations refer to the full names presented in Table S5.

In order ascertain the basis for variation in peroxidase (POD) activity (Figure 1D), we mined for POD-related genes from the DEGs and detected 13 transcripts annotated as POD [EC:1.11.1.7] (Table S5). Three transcripts (*GSBRNA2T00009862001, GSBRNA2T00078663001* and *GSBRNA2T00135615001*) were exclusively expressed GX74, where their expressions increased after cold-stress treatment. Furthermore, six other transcripts (*GSBRNA2T00121586001, GSBRNA2T00031824001, GSBRNA2T00136179001, GSBRNA2T00017367001, GSBRNA2T00055682001* and *GSBRNA2T00130723001*) exhibited higher expressions in GX74 as compared to XY15 after cold-stress treatment (Figure 6; Table S5). Some POD transcripts (*GSBRNA2T00087202001*) were upregulated in both genotypes in response to cold-stress treatment. These expression changes confirm the POD activity (Figure 1D). Additionally, 14 glutathione S-transferase (GST) transcripts were also highly upregulated in GX74 after cold treatment. Their expressions were higher in GX74 as compared to XY15. Whereas, in case of XY15, two ROS-scavenging-related genes, i.e., CAT1 (*GSBRNA2T00126684001*) and superoxide dismutase (SOD, *GSBRNA2T00154487001*), were exclusively expressed (downregulated) in response to cold-stress treatment. These changes are also consistent with the glutamate accumulation in rapeseed siliques after cold-stress treatment. Thus, our results indicate that cold-stress treatment increased antioxidant activity in GX74, but reduced expressions of CAT1, SOD, and POD transcripts caused cold sensitivity in XY15.

Since flavonoids also act as antioxidants [26] and DEGs were also enriched in the flavonoid biosynthesis pathway, we explored the expression changes in flavonoid biosynthesis genes in both genotypes under cold-stress treatment. Ten genes (*GSBRNA2T00105139001, GSBRNA2T00157050001, GSBRNA2T00067521001, GSBRNA2T00009809001, GSBRNA2T00043435001, GSBRNA2T00024512001, GSBRNA2T00090338001, GSBRNA2T00124629001, GSBRNA2T00123457001,* and *GSBRNA2T00109589001*) showed exclusive expressions (upregulation) in GX74 in response to cold treatment (Figure 6; Table S5). Whereas, only one trans-cinnamate 4-monooxygenase was upregulated in XY15 in response to cold stress. Thus, increased flavonoid biosynthesis genes is an indication of ROS scavenging.

**Phytohormone and MAPK signaling pathways are differentially regulated in XY15 and GX74 siliques in response to cold stress**

**Figure 7:**
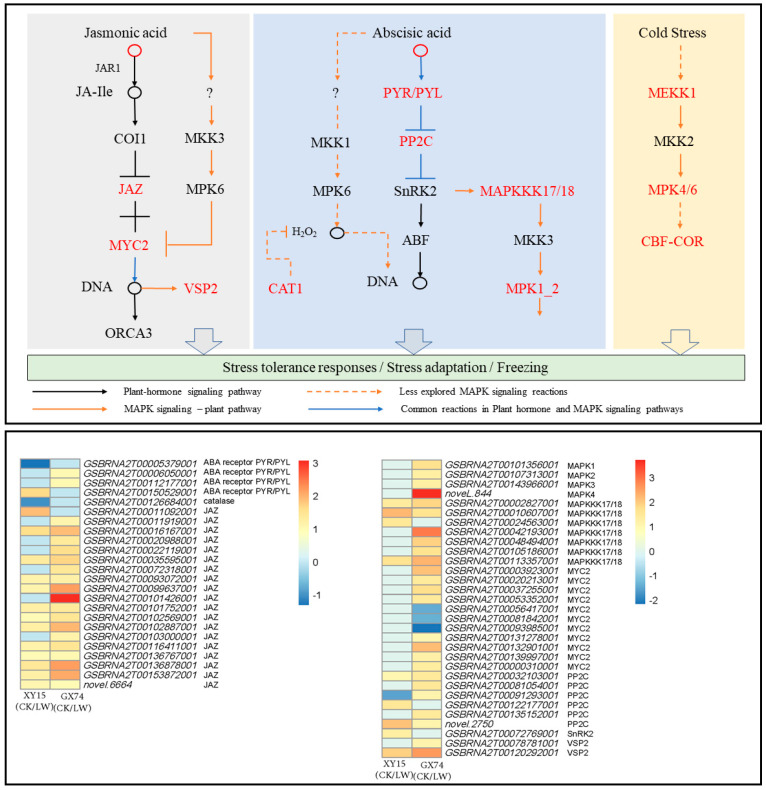
Expression changes in plant-hormone signaling and MAPK signaling—plant pathways in XY15 (cold-sensitive) and GX74 (cold-tolerant) rapeseed siliques—in response to cold treatment. The pathways are drawn according to the respective reference pathways in KEGG pathway database [25]. The heatmap was drawn in TBtools [27] using the log2 foldchange values of differentially expressed genes enriched in these two pathways. CK and LW represent control and cold treatments. The gene abbreviations and other genes refer to full names presented in Table S5.

The metabolome profiles of XY15 and GX74 indicated the accumulation of phytohormones, i.e., JA, Jasmonoyl-L-Isoleucine, N6-Isopentenyladenine, and ABA, in GX74 and reduced accumulation of N6-Isopentenyladenine and ABA in XY15 in response to cold-stress treatment. The reduced ABA accumulation in XY15LW is consistent with the downregulation of an abscisic acid receptor PYR/PYL (GSBRNA2T00005379001) and upregulation of four protein phosphatase 2C (PP2C, *GSBRNA2T00122177001, GSBRNA2T00122885001, novel.2750,* and *GSBRNA2T00032103001*). On the contrary, two PYR/PYLs (*GSBRNA2T00006050001* and *GSBRNA2T00112177001*) were highly upregulated, indicating increased ABA perception. These observations suggest that ABA biosynthesis is increased in response to cold-stress treatment, where higher ABA is detected in GX74 as compared to XY15 as evident from PYR/PYL expressions (Figure 7; Table S5). As observed in the metabolome profiles, the JA content in XY15LW was higher than that of GX74LW (Table S2), and the relatively higher FPKM values of the Jasmonate-ZIM-domain-containing proteins (JAZs) clearly correlates with it. Similarly, 10 MYC2 TFs were solely expressed in GX74, where seven of these were upregulated in response to cold stress. Thus, these observations indicate that in cold-treated rapeseed siliques in XY15, the JAZ expressions, which are relatively higher than in GX74, cause no release of downstream TFs, e.g., MYC2, thus controlling several downstream processes, i.e., secondary metabolite biosynthesis. This is also consistent with higher flavonoid biosynthesis genes’ expressions.

In the case of MAPK signaling, the ABA-related observations presented above are further supported by the upregulation of seven mitogen-activated protein kinase kinase kinase 17/18 (MAPKKK17/18) transcripts after cold-stress treatment in GX74, contrary to the increased expressions of only three of these transcripts in XY15. Further downstream, the exclusive downregulation of a catalase 1 (CAT1) gene (*GSBRNA2T00126684001*) in XY15 in response to cold treatment is consistent with the ABA accumulation and PYR/PYL genes’ expressions. Furthermore, the increased expressions of two vegetative storage protein 2 (VSP2) transcripts in GX74 in response to cold-stress treatment, validate the statements about the role of JA in cold-stress tolerance (Figure 7; Table S5).

Additionally, two genes enriched in the cold-tolerance section of the MAPK signaling pathway, i.e., mitogen-activated protein kinase kinase kinase 1 (MEKK1, *GSBRNA2T00060091001*) and mitogen-activated protein kinase 4 (MAPK4, *novel.844*), were highly upregulated in GX74 in response to cold-stress treatment. Similarly, the inducer of CBF expression 1 (ICE1, *GSBRNA2T00001133001*) was only expressed in GX74. This gene is a cold-responsive gene and increases cold tolerance [28]. Additionally, we observed that a relatively higher number of DREB transcripts (CBFs) were upregulated in GX74 (80) as compared to XY15 (44) after cold stress (Figure 7; Table S5). These expression changes indicate that cold stress induces MEKK1 and MAPK4 expressions, which activate the CBF-COR pathway for cold tolerance in GX74. Taken together, these results imply that JA and ABA signaling play significant roles in cold-stress tolerance in rapeseed. Moreover, the differential regulation of these hormone signals as well as MAPK signaling cascade related to salt stress renders higher cold tolerance to GX74.

**Expression changes in fatty acid metabolism/biosynthesis pathway**

The metabolome profiles showed increased accumulation of metabolites classified as lipids; therefore, we explored expression changes in fatty acid biosynthesis as well as fatty acid metabolism pathways. Fourteen genes enriched in these two pathways were differentially expressed. Only three, i.e., palmitoyl-protein thioesterase (upregulated), long-chain acyl-CoA synthetase (downregulated), and acetyl-CoA carboxylase biotin carboxyl carrier protein (downregulated) were expressed in XY15CK vs. XY15LW. The long-chain acyl-CoA synthetase transcripts were downregulated, whereas others, i.e., acyl-coA oxidase, acyl-coA dehydrogenase, acetyl-CoA carboxylase biotin carboxyl carrier protein, 17beta-estradiol 17-dehydrogenase/very-long-chain 3-oxoacyl-CoA reductase, enoyl-CoA hydratase/3-hydroxyacyl-CoA dehydrogenase, acetyl-CoA carboxylase/biotin carboxylase 1, and malonyl-CoA/methylmalonyl-CoA synthetase, were upregulated in response to cold-stress treatment in GX74 (Table S5).

**Expression changes in transcription factors**

**Figure 8:**
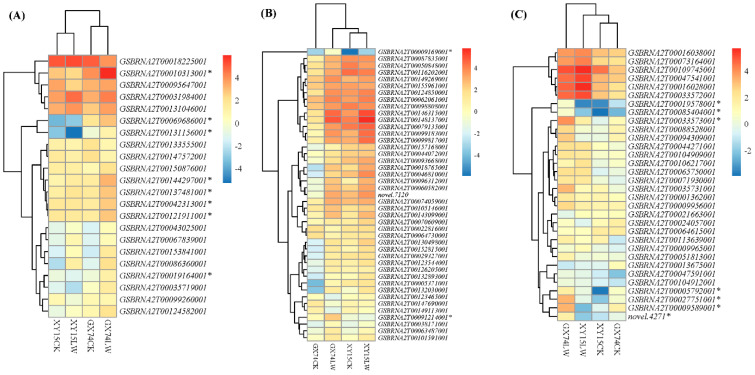
Heatmap of major transcription factors (family named on top of each heatmap) based on the log2-transformed mean value of fragments per kilobase of exon per million fragments mapped among the twelve samples (2 contrasting genotypes (XY15 and GX74) × 2 conditions (before cold treatment, CK; and cold treatment, LW) × 3 biological replicates, 1,2,3) mapped from rapeseed pods before and after cold stress. XY15 and GX74 are low-temperature-sensitive and -tolerant, respectively. (**A**). bZIP. (**B**). WRKY. (**C**). NAC. * Potential candidate genes responsible for the differential response in the two genotypes.

Several transcription factor (TF) families have been reported to regulate cold responses in plants including bZIP in *B. rapa* [29], WRKY in *Eucalyptus globulus* [30], NAC in *Oryza sativa* [31], and *Malus domestica* [32], among others. To this regard, we found that TFs belonging to 15 families were differentially expressed in the two genotypes under the influence of cold-stress treatment (Figure 5A–C). We observed the upregulation of eight bZIP TFs (*GSBRNA2T0010313001*, *GSBRNA2T00069686001*, *GSBRNA2T00131156001*, *GSBRNA2T00144297001*, *GSBRNA2T00137481001*, *GSBRNA2T00121911001,* and *GSBRNA2T00019164001*) (Figure 8A) and two WRKY TFs (*GSBRNA2T00009169001* and *GSBRNA2T00091214001*) in GX74 after cold-stress treatment (Figure 8B). Whereas seven NAC TFs (*GSBRNA2T00019578001*, *GSBRNA2T00085404001*, *GSBRNA2T00033573001*, *GSBRNA2T00005792001, GSBRNA2T002751001, GSBRNA2T00009389001* and *novel4271*) (Figure 8C) were weakly expressed in XY15 independent of the treatment, they were strongly induced by cold stress in the GX74 genotype, indicating the possibility of their involvement to cold tolerance in the GX74 genotype (Figure S5; Table S6). These expression trends clearly indicate that a large number of TFs take part in the cold-stress response in rapeseed, and the higher expressions of these TFs, particularly bZIP, WRKYs, NAC, MYB, bHLH, and others, play a role in cold-stress tolerance in GX74.

## 3. Discussion

Low temperature is a major environmental factor, which not only limits the plant growth, development, and productivity [33], but also poses a threat to food and nutrition security. To ensure the optimal growth and productivity of rapeseed cultivated along the middle and lower reaches of Yangtze River, which usually experience cold stress below 0 °C, we explored the key mechanisms that help rapeseed to withstand cold stress.

### 3.1. Higher Saccharides and Fatty Acids Biosynthesis Enables GX74 to Tolerate Cold Stress

More than five decades of research have shown that increased amounts of soluble sugars accumulate after cold stress [34]. Especially, the role of sugars in protecting cellular membranes under stress conditions is relatable to our results [35]. The results that TSS content increased in the cold-stress-treated siliques of both varieties indicates that similar mechanism is present in rapeseed (Figure 1C; Table S3). The observations that GX74 showed better growth (as observed by silique length (Figure 1A)) and had relatively higher TSS and saccharide contents as compared to XY15 are consistent with the earlier report that all sugars (fructose, glucose, sucrose, raffinose, etc.) had strong correlation with freezing tolerance in Vitis genotypes with contrasting freezing tolerance [36]. Moreover, an earlier study on coffee genotypes with different cold sensitivities reported higher expression of sugar contents and respiratory enzyme activities [37]. Particularly, the better growth of GX74 and respective higher expressions of HKs and PGM is consistent with the earlier reports on *Jatropha curcas* [38] and Arabidopsis [39]. Additionally, the XYL4 gene has been previously highlighted as an important gene in abiotic stress tolerance in alfalfa [40]; thus, it may also function similarly in rapeseed. Similarly, the higher expressions of UDP glucose 6-dehydrogenase and PGs in GX74 after cold treatment confirm that cold-stress tolerance in rapeseed is similar to winter barley [41] and rice [42]. Finally, exclusive expressions (upregulations) of genes regulating the biosynthesis of α-D-glucose, α-D-glucose 6p, β-D-glucose, β-D-glucose 6p, and β-D-fructose 6p, such as PEC, GalAG, UDPglucose 6-dehydrogenase, and PL, in GX74 after cold-stress treatment (Table S5) confirms that cold tolerance is due to increased sugar contents [43].

Likewise, the increased accumulations of free fatty acids such as erucic acid, ricinoleic acid, elaidic acid, palmitic acid, linoleic acid, stearic acid, and octanoic acid in response to cold-stress treatment is consistent with an earlier report on *Capsella bursa pastoris* [44]. To this regard, the higher expression of multiple genes involved in fatty acid biosynthesis supplements the proposition that these metabolites play roles in cold-stress tolerance. The results that malonyl-CoA synthetase expression increased after cold stress are consistent with the earlier findings that its overexpression in Arabidopsis resulted in enhanced cold tolerance [45]. The same study indicated that malonyl-CoA synthetase activates MAPK signaling in plants, which is consistent with our findings (Figure 7). Thus, these results indicate a similar mechanism of cold tolerance present in rapeseed. Further characterization of key players of this metabolism can shed light on the role of individual genes involved.

### 3.2. Increased Expressions of ROS Scavenging and Flavonoid Biosynthesis Genes Enable GX74 to Tolerate Cold Stress

The plant antioxidative system comprises enzymatic and non-enzymatic antioxidative components [46]. Cold stress induces the production of ROS in different organelles of plant cell, i.e., chloroplast, mitochondria, peroxisome, and apoplast [47]. These ROS damage molecular and cellular components as a result of the oxidation of biomolecules such as lipids, carbohydrates, proteins/enzymes, and DNA [48,49]. The antioxidative system acts as a shield against the cold stress and thus protects plant cells and allows their normal growth [49]. Our results that POD activity significantly increased in GX74 after cold treatment (Figure 1D) and the consistent upregulation of POD transcripts (Figure 6) imply that the cold tolerance in this genotype is due to increased antioxidant activity. A cold-responsive peroxidase (CbRCI35) from *C. bursapastoris* regulated ROS homeostasis and enhanced cold tolerance in tobacco [50]. Our proposition that GX74 had higher antioxidative potential is further confirmed by the increased expressions of GPX, GST, and SOD transcripts in GX74 after cold treatment. Contrarily, the reduced expression of GST, SOD, POD, and CAT in XY15 after cold treatment clearly indicate its inability to scavenge ROS, which makes it cold-susceptible. These observations are consistent with earlier reports on tomatoes [49], cotton [51], and Juglans regia [49]. Furthermore, the results that flavonoid biosynthesis genes were highly expressed after cold treatment in GX74 (Table S5) indicate that this genotype also employs non-enzymatic quenching of ROS, thus shielding it against cold stress, similar to other plants [26,52]. Taken together, higher expressions of POD, SOD, GST, GPS, and flavonoid biosynthesis genes enable GX74 to tolerate cold stress.

### 3.3. Reduced Expressions of Photosynthesis, Antenna Proteins, and Chlorophyll Biosynthesis Are Related to Cold Sensitivity in XY15

Cold severely affects the chloroplast, which leads to changes in chlorophyll biosynthesis, ultimately creating an imbalance in PSI and PSII. These changes are also associated with the alterations in antenna proteins [53]. Our results that PSI (LhcA1, LhcA3, and LhcA4) and PSII (LhcB1, LhcB2, LhcB3, LhcB4, and LhcB6) were downregulated in XY15LW (Figure 5) clearly suggest that cold stress significantly affected the light-harvesting complex (LHC) in XY15. Such a mechanism is reported in conifers, where the organization of the photosynthetic apparatus changed the number of functional PSI and PSII LHCs [54]. It is important to mention that the expression changes in a larger number of transcripts associated with PSII also indicate that PSII damage under cold stress is more pronounced than that of PSI [55]. These changes have also been previously associated with increased xanthophyll contents [56]. The XY15LW siliques turned yellow and carotenoid biosynthesis pathways were also significantly enriched; therefore, we conclude that changes in LHC are related to xanthophylls in rapeseed [14]. The observations that CHLase, protoporphyrinogen, glutamate-1-semialdehyde 2,1-aminomutase and petF, petH, psaN, Psb27, and PSI subunits II, VI, and X were downregulated in XY15LW (Figure 4) validate that cold stress disturbs both the chlorophyll biosynthesis and photosynthesis and thus XY15 siliques remain shorter. Previously, Kubien et al. [57] reported that cold-induced changes in growth were associated with the slowing of photosynthesis and low metabolic activities. D-Fructose-1,6-biphosphate was a prominent metabolite enriched in photosynthesis and its related pathways (Table S3). It has been implicated in a decreased net photosynthetic rate, ribulose-1,5-bisphosphate, and soluble sugar content, which affected the stem diameter, dry weight, and size in cold-sensitive tomato seedlings [58]. Thus, our results indicate that, similar to earlier reports, the changes in TSS/saccharides are associated with the variation in photosynthesis in rapeseed. Thus, our results complement the earlier reports that a more frequent damage is caused to PSII in response to cold stress. Taken together, the comparative transcriptome profiles of the XY15 and GX74 siliques under cold-stress treatment indicate that disturbances in antenna proteins, photosynthesis, and chlorophyll biosynthesis cause cold sensitivity in XY15.

### 3.4. ABA, JA, and MAPK Signaling Are Related to the Contrasting Cold Tolerance in XY15 and GX74

Complex signaling cascades involving phytohormones (particularly JA and ABA) induce changes in cold-responsive genes’ expressions that enable plants to tolerate cold stress [59]. Both the transcriptome and metabolome results complemented each other that higher ABA contents in GX74 were consistent with PYR/PYL expression. The PYR/PYLs are the ABA receptors and a previous study indicated that their higher expressions are related to cold tolerance in Arabidopsis [60]. PYR/PYLs recruit PP2Cs and release SnRK2s from sequestration with PP2Cs. The released SnRK2s then activate downstream genes to adapt the stress. Considering the layout of the ABA signaling pathway in MAPK signaling—plant (K04016), the higher expressions of MAPKKK17/18 in GX74 after cold treatment indicate that the ABA signal is perceived, and downstream genes are activated. This is consistent with the known interaction of ABA and MAPKKK17/18 that the former is required for the expression of the latter [61]. Therefore, our results indicate that, in GX74, relatively higher ABA is detected by the PYR/PYLs, which then allow the increased expression of MAPKKK17/18 through a signaling cascade, and GX74 adapts well to the cold stress as compared to XY15. However, GX74’s cold tolerance can also be due to another signaling cascade, i.e., MEKK1-MKK2-MAPK3/4, in the MAPK signaling pathway. The exclusive expressions of MEKK1, MAPK4, and ICE1 and upregulation of a large number of CBF transcripts in GX74 as compared to XY15 indicate the activation of the MEKK1-MKK2-MAPK3/4 cascade, which then activates the CBF-COR pathway in GX74 siliques (but absent in XY15) thus enabling it to tolerate cold stress like Arabidopsis [62]. Earlier studies reported that ABA improves photosynthesis by stabilizing photosystem II in cold-tolerant *Cynodon dactylon* [63]; therefore, it is possible that a similar mechanism exists in GX74. These statements are supported by the expression changes in photosynthesis and antenna proteins in both genotypes (Table S5).

Jasmonic acid’s increased concentration is attached to F-box protein (COI1) and promotes ubiquitination of JAZ repressor proteins and degrades them. This degradation derepresses several TFs and allows JA-driven responses [64]. Our results that JA content increased in both XY15 and GX74 indicates its role in cold stress in rapeseed (Table S2). However, the relatively lower FPKM values of JAZ proteins in GX74LW as compared to XY15LW indicate higher JAZ degradation in the former genotype’s siliques and the release of MYC2 TFs in GX74 after cold stress corresponds to this change. The MYC2s in turn contribute to the cold tolerance in GX74, similar to *Poncirus trifoliata* [65] and apple [66]. The VSP2 gene has been previously reported as a MYC-regulated gene [67] as well a cold-responsive gene [68]. Therefore, it is understandable the JA signals activate MYC2s in GX74 after cold stress and the expressions of cold-responsive genes are increased, which make this genotype cold-tolerant and XY15-susceptible.

Taken together, based on the accumulation patterns of ABA and JA, and expression changes in signaling of these hormones as well as the MAPK signaling pathway, we conclude that both JA and ABA promote cold-stress tolerance by activating signaling cascades.

### 3.5. Differences in Expressions of TFs Are Related to the Contrasting Cold Tolerance in XY15 and GX74

Exposure to cold stress triggers expression changes in many TFs, which further assist plants to tolerate the stress. The results that a large number of TFs belonging to 15 families were expressed in the studied treatments indicate their intensive roles in cold stress. Particularly, the higher expressions of bZIP, WRKY, NAC, MYB, bHLH, and other TFs in GX74 as compared to XY15 after cold stress indicate that large-scale transcriptional changes drive cold tolerance. bZIP TF has been previously implicated in cold tolerance in Japonica rice at the seedling stage as well as the reproductive stage by interacting with the sugar transport pathway [69,70]. We observed that TSS and saccharide contents increased in GX74 after cold stress (Figure 1; Table S3); therefore, a possible link between bZIP TF expression and sugars can be expected. However, WRKYs (WRKY46, WRKY34, and WRKY 94) have been characterized in B. napus [71], Arabidopsis [72], and rice [73] as against cold stress. Our observations that WRKY1, WRKY2, WRKY22, and WRKY 33 were highly upregulated in GX74 after cold stress indicate their roles in cold tolerance. WRKYs are also activated by ABA signaling [74]; therefore, the higher ABA content, subsequent signal transduction, and activation of the CBF-COR pathway, together with the higher WRKYs expressions, indicate an interplay of these genes. This statement is based on the above-mentioned WRKY46’s role in B. napus in cold-stress tolerance and the fact that WRKY22 and WRKY33 belong to the same phylogenetic group [75]. Likewise, bHLHs also take a part in cold tolerance by interacting with COR and ROS pathways [76]. Other than these TFs, the MYBs also regulate cold tolerance by increasing flavonoid accumulation [77], which is consistent with our findings that flavonoid accumulated in higher quantities in GX74 and also that the MYBs were highly expressed in GX74 after cold treatment. Taken together, we conclude that increased expressions of TFs (particularly MYB, WRKY, bZIP, and bHLH) in GX74 as compared to XY15 are related to cold-stress tolerance.

## 4. Materials and Methods

### 4.1. Plant Materials and Treatments

Two rapeseed (*Brassica napus* L.) genotypes, i.e., XY15 and GX74, were obtained from the Key Laboratory of Crop Epigenetics Regulation and Development in Hunan Province, Hunan Agricultural University, Hunan, China. XY15 is a conventional rapeseed variety. GX74 is a mutant developed by ethyl methylsulfonate mutagenesis of XY15 and the GX74 seeds used were obtained by seven successive rounds of self-breeding.

Pilot experiments have shown that XY15 and GX74 genotypes are cold-sensitive and cold-tolerant, respectively. The healthy and fully developed seeds of the two genotypes were selected and germinated on two wet filter papers in a Petri dish in a light incubator at 22 ± 1 °C. After germination, uniform seedlings were transferred to a 1.5-gallon pot containing a mixture of vermiculite and soil. After outdoor cultivation, the seedlings were transferred to the growth chamber (temperature 20 ± 2 °C, 16 h of light and 8 h of dark, and light intensity of 6000 Lx) and allowed to grow until flowering. A total of 120 plants with homogenous growth were selected, and the flowers that opened that day were labeled and hand-pollinated. Three days later, the plants were divided into two groups, i.e., 60 plants were used as control (CK), while the other 60 plants were stressed with low temperature (−2 °C) in the dark for 2 h.

After the application of cold stress for 2 h (designated LW), the LW plants were returned to the growth chamber for normal growth. Silique samples were collected from the triplicate LW plants upon their return to the growth chamber, then they were immediately frozen in liquid nitrogen and stored in the refrigerator at −80 °C until processed further. All the experiments were conducted in the experimental base of Hunan Agricultural University, Hunan, China (28010′49″ N, 11034′39″ E). The experimental area is located in a subtropical monsoon climate with an average annual temperature of 17 °C, and an average annual frost-free period, rainfall, and sunshine duration of 274 days, 1422 mm, and 1663 h, respectively.

### 4.2. Physiological Index Measurements and Statistical Analysis

The silique lengths of the CK and LW samples were measured with the help of a meter rule. The sample total soluble sugar (TSS) contents were determined with a Plant Soluble Sugar Content Assay Kit (BC0030, Beijing Soleibao Technology Company, LTD, Beijing, China). The POD activity was determined with a Peroxidase Assay Kit (A08-3-1, Nanjing Jiancheng Bioengineering Institute, Nanjing, China). Data obtained were subjected to a two-tailed t-test (5% probability) in Microsoft Excel 2019^®^ (www.microsoft.com).

### 4.3. Ultra-High-Performance Liquid Chromatography-Mass Spectrometry and Data Analyses

Three grams of each of the 12 samples from the two rapeseed genotypes were crushed into powder after vacuum freeze-drying using a mixer mill MM400 with zirconia beads with 15 mm for 1.5 min at 30 Hz. Sample powder of 100 mg was extracted overnight at 4 °C in 1.0 mL 70% aqueous methanol and centrifuged for 10 min at 10,000 × g for the absorption of the extractives. The extractives were further filtered and transferred to a new tube for an ultra-high-performance liquid chromatography-tandem mass spectrometry (ULPLC-MS/MS) analysis. The analyses were conducted by the MetWare Biotechnology Co. Ltd. (Wuhan, China) following protocols published by Wen et al. [78].

Widely untargeted metabolites profiling was conducted with a self-built database by MetWare Biotechnology Co. Ltd. (Wuhan, China). The metabolites were qualitatively analyzed with secondary spectrum information. The metabolites detected were quantified by a multireaction monitoring mode analysis by triple quadruple-bar mass spectrometry. Data quality and reproducibility were assessed by Pearson correlation and principal component analysis (PCA). The differentially accumulated metabolites (DAMs) were detected by application of partial least squares-discriminant analysis (PLSDA) with a threshold of log2FC ≥ 1 and variable importance of the projection (VIP) ≥ 1 [79].

### 4.4. Transcriptome Sequencing and Data Analyses

Triplicate cold-treated rapeseed siliques samples (XY15 and GX74) stored in a −80 °C refrigerator and their respective controls were used to isolate total RNAs using a Tiangen RNAprep Pure Plant Kit (Tiangen, China) according to the manufacturer’s instructions with three biological repeats. The RNA quality was measured by NanoDrop (Thermo Scientific, Waltham, MA, USA). The cDNA of the 12 samples (XY15CK1-3, XY15LW1-3, GX74CK1-3, and GX73LW1-3) were prepared following the manufacturer’s protocols (NEB Next RNA Library Prep Kit). The quality and quantity of the cDNA libraries for sequencing were evaluated with the Agilent 2100 bioanalyzer system (Agilent Technologies, Palo Alto, CA, USA). The cDNA libraries were sequenced by Illumina paired-end sequencing technology with a read length of 100 bp on an Illumina HiSeq 2000 instrument by MetWare Biotechnology Co., Ltd., (Wuhan, China).

To obtain high quality reads, the raw reads were filtered by removing low-quality reads in the FastQC program with the default settings (http://www.bioinformatics.babraham.ac.uk/projects/fastqc/; accessed on 13 May 2022) and the clean data were then subjected to further analyses. The high-quality raw reads of each library were mapped to the rapeseed reference genome “Darmor-bzh” [1] with HISAT2 software with default settings [80]. Raw counts of genes were determined by using featureCounts [81]. The detected genes were annotated to a translation of the European Molecular Biology Laboratory (TrEMBL) [82], non-redundant (Nr) [83], EuKaryotic Orthologous Group (KOG) [84], gene ontology (GO) [85], protein families (Pfam) [86], SwissProt [87], Kyoto Encyclopedia of Genes and Genomes (KEGG) [88], and Plant transcription factors database (PlantTFDB) 3.0 [89]. Thresholds of log2 foldchange (log2FC) ≥ 1 and false discovery rate (FDR) ≤ 0.05 [79] were used to detect differentially expressed genes (DEGs) with the DESeq2 package in R [90]. Pearson correlation and principal components analyses were conducted in R with the packages corrplot and ggbiplot, respectively (R Core Team, 2018).

To validate the data of RNA-seq, 20 DEGs showing interesting expression profiles related to the discussed pathways were selected for quantitative reverse-transcription polymerase chain reaction (qRT-PCR). The qRT-PCR reactions were performed according to the manufacturers’ protocols (TaKaRa Biotech Dalian, China; code: DRR037A) and Tiangen Biotech (Dalian, China; code: FP204). All reactions were in three technical and three biological replicates in a Roche HOLD CYCLE LightCycler 480 II (Roche, Mannheim, Germany). The rapeseed Actin gene was used as the housekeeping gene. The DEGs and housekeeping gene primers used are listed in Table S1. The relative expression levels were computed according to the 2^−∆∆Ct^ method [91]. The data obtained were subjected to correlation analysis together with RNA-seq data with the help of Microsoft Excel^®^ 2019 (www.microsoft.com).

## 5. Conclusions

The combined transcriptome and metabolome analyses supplemented with physiological analyses of the siliques of the two B. napus varieties are presented in this study. We conclude that the differences in the XY15 (cold-sensitive) and GX74 (cold-tolerant) varieties can be mainly related to the differential accumulation and regulation of saccharides. Genes enriched in several key pathways governing the accumulation of sugars were found associated with the observed cold sensitivity differences. We found that, in XY15, the genes related to chlorophyll biosynthesis, antenna proteins, and photosynthesis pathways were downregulated, implying that cold stress significantly disturbs photosynthetic potential in XY15 as compared to GX74. On the contrary, higher expressions of flavonoid biosynthesis and ROS scavenging genes, together with increased POD activity, are associated with cold-stress tolerance in GX74. The differential content of the two phytohormones, i.e., ABA and JA, generates signaling cascades that ultimately affect the expressions of downstream genes and produce cold-stress responses in both genotypes. We also found that the MAPK signaling pathway and CBF-COR pathway are related to the cold-stress tolerance in rapeseed.

## Data Availability

The raw RNA-seq data have been submitted to NCBI SRA under the project number: PRJNA915808. The data will be released upon publication of this manuscript.

## Supplementary Materials

The following supporting information can be downloaded at: https://www.mdpi.com/article/10.3390/ijms232113546/s1.
