# Supplementary material for: Combined Transcriptome and Metabolome Profiling Provide Insights into Cold Responses in Rapeseed (Brassica napus L.) Genotypes with Contrasting Cold-Stress Sensitivity
Source: Int J Mol Sci. 2022 Nov 4;23(21):13546. doi: 10.3390/ijms232113546 (PMC9657917; doi:10.3390/ijms232113546)
Supplement: Supplementary file 1 [file ijms-23-13546-s001.zip › ijms-1996176-Figure S2.pdf]

International Journal of
Molecular Sciences

MDPI

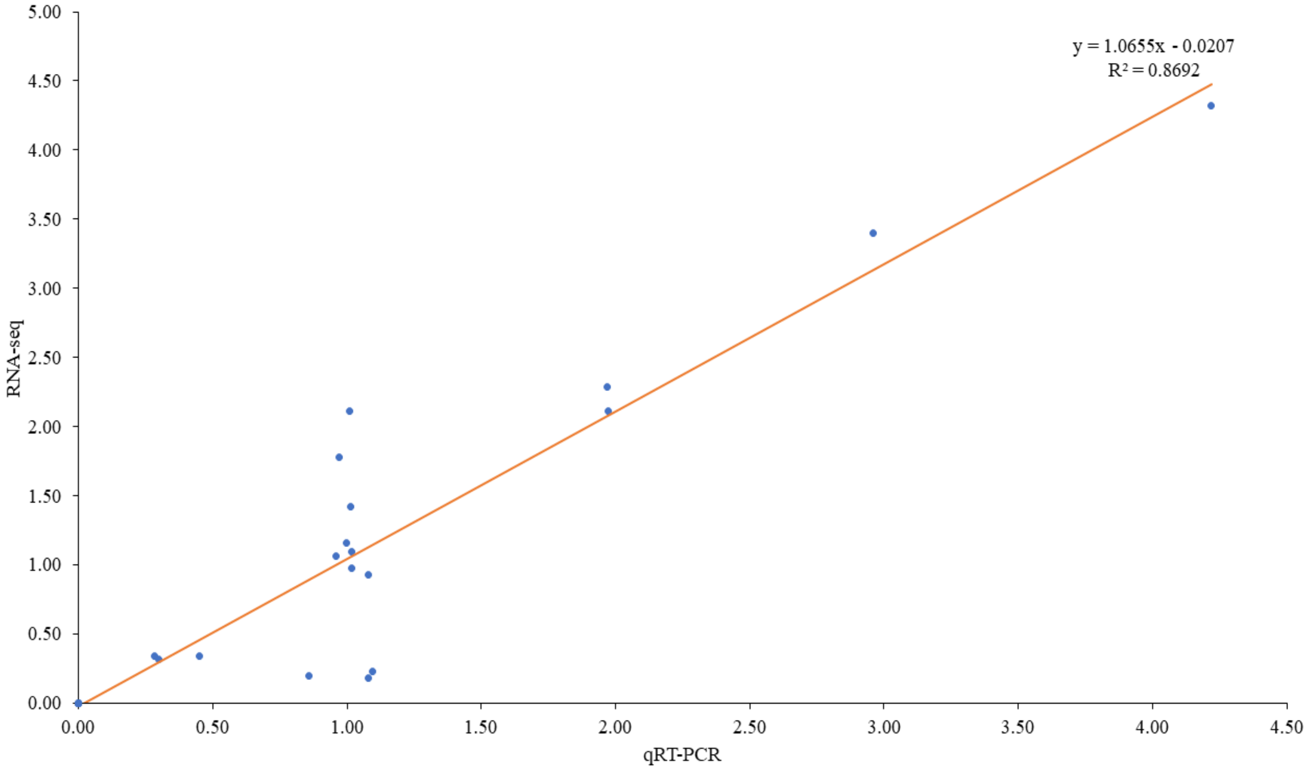

Supplementary Figure S2. Validation of RNA-seq data by real time quantitative PCR (qRT-PCR) in the two contrasting genotypes (XY15 and GX74, susceptible and tolerant) with siliques before (CK) and after cold stress treatment (LW).
