# Supplementary material for: Combined Transcriptome and Metabolome Profiling Provide Insights into Cold Responses in Rapeseed (Brassica napus L.) Genotypes with Contrasting Cold-Stress Sensitivity
Source: Int J Mol Sci. 2022 Nov 4;23(21):13546. doi: 10.3390/ijms232113546 (PMC9657917; doi:10.3390/ijms232113546)
Supplement: Supplementary file 1 [file ijms-23-13546-s001.zip › ijms-1996176-Figure S3.pdf]

International Journal of *Molecular Sciences*

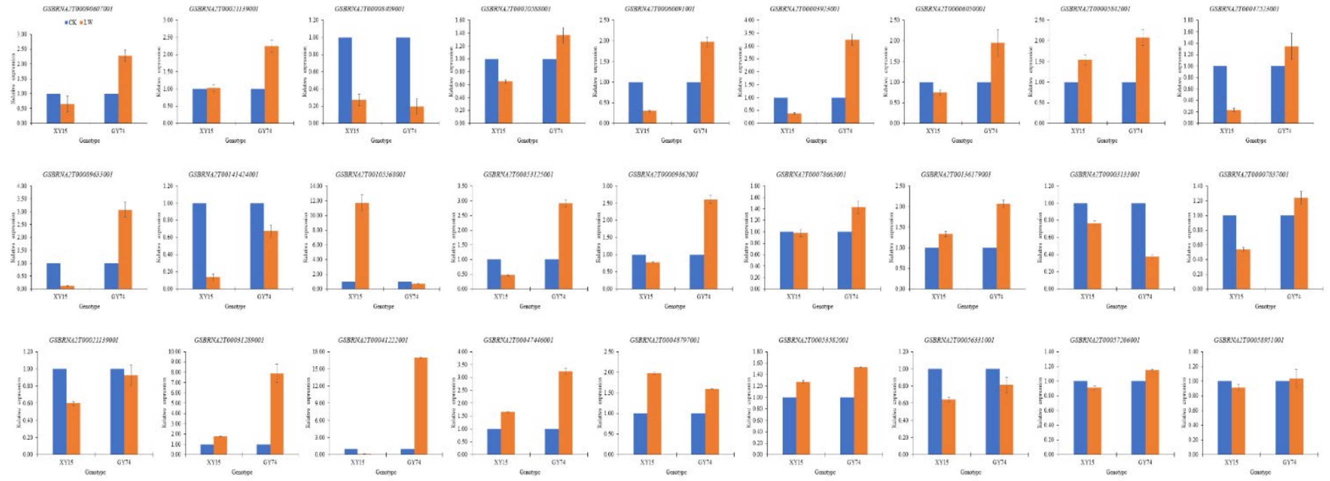

Supplementary Figure S3. Validation of RNA-seq data by real time quantitative PCR in the two contrasting genotypes (XY15 and GX74, susceptible and tolerant) with siliques before (CK) and after cold stress treatment (LW). Genes and their pathways are shown in Table S1.
