# Supplementary material for: Combined Transcriptome and Metabolome Profiling Provide Insights into Cold Responses in Rapeseed (Brassica napus L.) Genotypes with Contrasting Cold-Stress Sensitivity
Source: Int J Mol Sci. 2022 Nov 4;23(21):13546. doi: 10.3390/ijms232113546 (PMC9657917; doi:10.3390/ijms232113546)
Supplement: Supplementary file 1 [file ijms-23-13546-s001.zip › ijms-1996176-Figure S4.pdf]

International Journal of
Molecular Sciences

MDPI

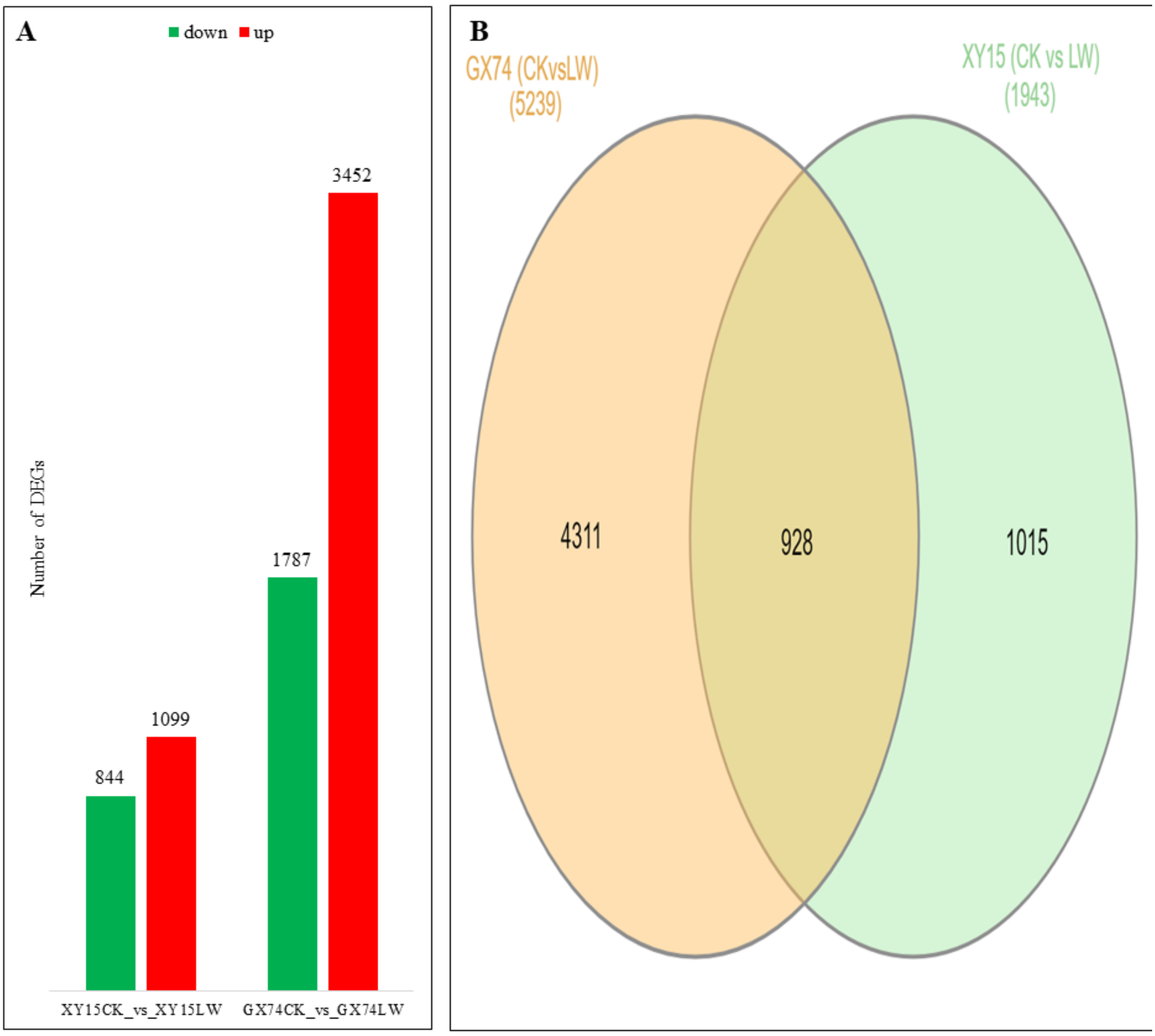

Supplementary Figure S4. (A) Differentially expressed genes (DEGs) of two contrasting genotypes of rapeseed under before and after cold treatment (CK and LW).**(B)** Venn diagram representing common and variety specific DEGS.
