# Supplementary material for: Combined Transcriptome and Metabolome Profiling Provide Insights into Cold Responses in Rapeseed (Brassica napus L.) Genotypes with Contrasting Cold-Stress Sensitivity
Source: Int J Mol Sci. 2022 Nov 4;23(21):13546. doi: 10.3390/ijms232113546 (PMC9657917; doi:10.3390/ijms232113546)
Supplement: Supplementary file 1 [file ijms-23-13546-s001.zip › ijms-1996176-Figure S5.pdf]

International Journal of
Molecular Sciences

MDPI

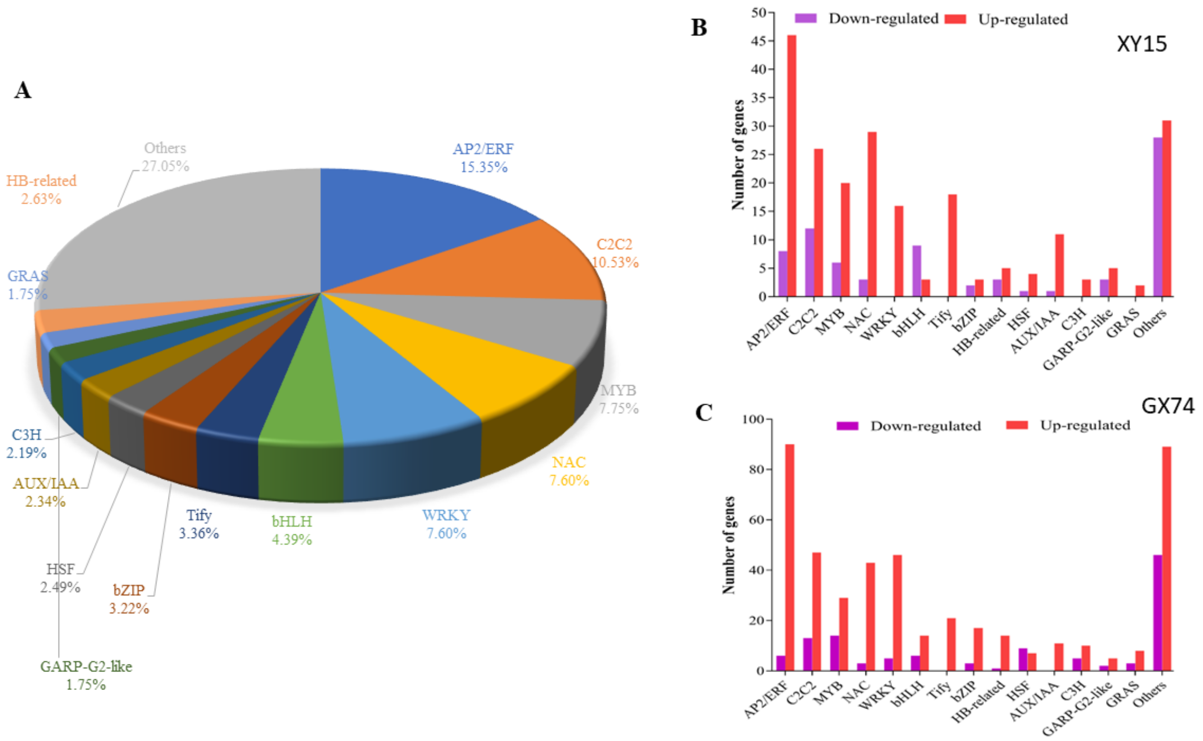

Supplementary Figure S5. Summary of the number of expressed transcription factors in XY15 and GX74 siliques before (CK) and after cold stress treatment (LW). A) Pie chart of major transcription factor families and others. B) Extent of expression of major transcription factors in the cold sensitive genotype (XY15) at CK and LW. C) Extent of expression of major transcription factors in the cold tolerant genotype (GX74) at CK and LW. Down-regulation means expression reduced upon cold stress, while up-regulated means expression increased upon cold stress.
